# Immune checkpoint inhibitor-related Stevens-Johnson syndrome and toxic epidermal necrolysis: a retrospective analysis of 21 cases

**DOI:** 10.3389/fimmu.2025.1734346

**Published:** 2026-01-12

**Authors:** Tianshu Pu, Yaru Teng, Yuezhu Zhang, Meihong Da, Fei Wang

**Affiliations:** 1 Medical College, Southeast University, Nanjing, China; 2 Department of Dermatology, ZhongDa Hospital Southeast University, Nanjing, China

**Keywords:** cutaneous adverse reactions, dermatology, drug eruptions, ICIS, immune checkpoint inhibitors, immune-related adverse events, severe drug eruptions, Stevens-Johnson Syndrome

## Abstract

**Introduction:**

Stevens-Johnson syndrome (SJS) and toxic epidermal necrolysis (TEN) are rare but life-threatening severe cutaneous adverse reactions (SCARs) increasingly linked to immune checkpoint inhibitors (ICIs).

**Methods:**

We retrospectively analyzed 21 patients with ICI-related SJS/TEN treated at Zhongda Hospital, Southeast University, from 2019 to 2025.

**Results:**

The median latency from ICI initiation to onset was 28 days, most commonly following PD-1 inhibitors such as sintilimab and tislelizumab. Patients presented with diffuse erythema, blistering, erosions, and frequent mucosal involvement. All discontinued ICIs and received systemic corticosteroids; some additionally received intravenous immunoglobulin (IVIG). The mean time to re-epithelialization was about 10 days, and mortality reached 14.3%, limited to TEN cases.

**Conclusion:**

ICI-related SJS/TEN, though rare, represents a serious immune-related adverse event that requires prompt recognition and early immunosuppressive therapy. Increased awareness and further studies are needed to clarify its mechanisms and guide management. Based on our findings, we recommend heightened vigilance for early mucocutaneous symptoms in patients receiving ICIs, prompt dermatology referral for suspected cases, and establishment of standardized reporting pathways to national pharmacovigilance systems to ensure rapid identification and pooled analysis of ICI-related SJS/TEN cases.

## 1 Introduction

Stevens-Johnson syndrome (SJS) and toxic epidermal necrolysis (TEN) are severe cutaneous adverse reactions (SCARs) characterized by extensive epidermal necrosis, blistering, and mucosal involvement. These disorders often have an abrupt onset and rapid progression, with high morbidity and mortality, underscoring the importance of early recognition and multidisciplinary management (1). Epidemiological studies indicate that the mortality rate is 4.8% for Stevens-Johnson syndrome (SJS), 19.4% for SJS-TEN overlap, and 14.8% for toxic epidermal necrolysis (TEN) (2). Since the approval of the first immune checkpoint inhibitor (ICI), the cytotoxic T-lymphocyte-associated antigen 4 (CTLA-4) antibody ipilimumab, in 2011, ICIs targeting PD-1, PD-L1, and CTLA-4 have revolutionized cancer therapy and significantly improved survival outcomes across multiple tumor types (3). However, their potent immune activation may also trigger immune-related adverse events (irAEs), among which cutaneous manifestations are the most frequent. While most dermatologic irAEs are mild and reversible, a small subset may progress to life-threatening SCARs such as SJS/TEN (4–6). With the expanding use of PD-1/PD-L1 inhibitors, increasing reports of ICI-related SJS/TEN have emerged in the literature. These cases may differ from classical drug-induced SJS/TEN in latency, clinical features, histopathology, and treatment response. Nevertheless, most published data are limited to individual case reports, and comprehensive analyses remain scarce. Therefore, our study retrospectively analyzed 21 patients with ICI-related SJS/TEN to summarize their clinical characteristics, laboratory findings, therapeutic approaches, and outcomes. By providing real-world evidence from a relatively larger cohort, this study aims not only to enhance clinical recognition and management of this rare but potentially fatal complication but also to contribute to broader awareness among clinicians and stakeholders and to support the improvement of post-marketing surveillance strategies for immune checkpoint inhibitors.

## 2 Methods

### 2.1 Study design and case identification

We reviewed the medical records of hospitalized patients diagnosed with SJS or TEN at Zhongda Hospital, Southeast University, between October 2019 and October 2025. Cases were screened for prior exposure to immune checkpoint inhibitors (ICIs), including PD-1/PD-L1 and CTLA-4 inhibitors. Only patients who had received ICIs before the onset of SJS/TEN were included in the analysis.

### 2.2 Inclusion and exclusion criteria

Patients were eligible if they developed clinically and histopathologically confirmed SJS/TEN after exposure to immune checkpoint inhibitors. To minimize confounding in causality assessment, we excluded individuals with recent exposure (within the preceding 8 weeks) to medications with a well-established, high intrinsic risk for inducing SJS/TEN—such as allopurinol, lamotrigine, carbamazepine, and sulfasalazine—because these agents could independently precipitate severe cutaneous adverse reactions. Patients with incomplete records or uncertain temporal associations between drug exposure and onset were also excluded.

### 2.3 Causality assessment

After patient selection, the causality between ICI exposure and SJS/TEN was systematically assessed using the Adverse Drug Reaction (ADR) Probability Scale proposed by Naranjo et al. (1981) (7). According to this scale, a score of ≥9 indicates a definite relationship, 5–8 probable, 1–4 possible, and ≤0 doubtful.

Severity AssessmentThe Severity-of-Illness Score for Toxic Epidermal Necrolysis (SCORTEN) was calculated for each patient to assess disease severity and predict mortality. The seven variables included in SCORTEN—age >40 years, presence of malignancy, heart rate ≥120/min, percentage of epidermal detachment ≥10%, serum urea >10 mmol/L (or BUN >28 mg/dL), serum glucose >14 mmol/L, and serum bicarbonate <20 mmol/L—were extracted for all patients, according to the original description by Bastuji-Garin et al. (2000) (8).

### 2.4 Data collection

For each patient, we collected demographic characteristics, clinical features, and laboratory findings upon admission, and evaluated the clinical course, including treatment, hospitalization duration, and clinical outcomes. When skin biopsies were performed, histopathological features consistent with SJS/TEN (such as full-thickness epidermal necrosis and subepidermal blistering) were recorded, although biopsy was not routinely conducted due to extensive skin detachment and patient severity. All laboratory parameters were derived from the initial measurements taken upon hospital admission.

Ethical Considerations This study was conducted in accordance with the principles of the Declaration of Helsinki. The study protocol was reviewed and formally approved by the Institutional Ethics Committee of Zhongda Hospital, Southeast University (Approval No. 2025ZDSYLL361-P01). As the research involved a retrospective analysis of anonymized medical records, the requirement for written informed consent was waived by the committee. Patient anonymity and data confidentiality were strictly maintained throughout the study. The report adheres to the STROBE guidelines for observational studies.

### 2.5 Statistical analysis

Descriptive statistical methods were applied, consistent with the observational and non-comparative nature of this case series. Continuous variables, including age, latency period, number of immune checkpoint inhibitor (ICI) cycles, laboratory parameters, and hospitalization duration, were summarized as medians with ranges due to non-normal distribution and the small sample size. Categorical variables, such as sex, primary malignancy type, comorbidities, clinical classification (SJS, SJS/TEN overlap, TEN), mucosal involvement, complications, histopathological features, and treatment modalities, were presented as frequencies and percentages. SCORTEN scores were calculated for each patient and summarized descriptively. No hypothesis testing or regression modelling was conducted because the objective of the study was to characterize clinical patterns rather than compare predefined groups or estimate associations. Missing data were rare and were handled by complete-case reporting without imputation. All statistical analyses and data tabulation were performed using IBM SPSS Statistics 26.0 software and reviewed independently by two investigators to ensure accuracy.

## 3 Results

### 3.1 Basic characteristics

A total of 21 patients with ICI-related SJS/TEN were included in this study, comprising 16 males and 5 females (**Table 1**). The age ranged from 49 to 82 years, with a median of 68 years. All patients had received ICI therapy for malignancies. The primary tumors included lung cancer (n = 9), gastric cancer (n = 5), esophageal cancer (n = 3), hepatocellular carcinoma (n = 2), bladder cancer (n = 1), and sigmoid colon cancer (n = 1); four patients had two concurrent malignancies. At admission, five patients were in partial remission, eight had disease progression, and eight had unevaluated therapeutic responses. Comorbidities included hypertension (n = 4), diabetes mellitus (n = 3), atrial fibrillation (n = 1), chronic bronchitis (n = 1), and hypothyroidism (n = 2). Two patients had a prior history of drug allergy (penicillin and cephalosporin).

**Table 1:** Baseline characteristics of 21 patients with immune checkpoint inhibitor-associated severe cutaneous adverse reactions.

Parameter	Classification	Value
Sex	male	16(76.2%)
	female	5(23.8%)
Age	year	68(range, 49-82)
Indication	lung cancer	9(42.9%)
	gastric cancer	5(23.8%)
	esophageal cancer	3(14.3%)
	hepatocellular carcinoma	2(9.5%)
	bladder cancer	1(4.8%)
	sigmoid colon cancer	1(4.8%)
Comorbidities	hypertension	4(19%)
	diabetes mellitus	3(14.3%)
	atrial fibrillation	1(4.8%)
	chronic bronchitis	1(4.8%)
	hypothyroidism	2(9.5%)
Latency Period	days	28 (range, 1-365)
Immunotherapy Regimens	Sintilimab (mono)	8(38.10%)
	Tislelizumab (mono)	4(19%)
	Camrelizumab(mono)	2(9.5%)
	Serplulimab(mono)	1(4.8%)
	Toripalimab(mono)	1(4.8%)
	Sintilimab + Bevacizumab	1(4.8%)
	Tislelizumab + Bevacizumab	1(4.8%)
	Adebrelimab	1(4.8%)
	Atezolizumab	1(4.8%)
	Ipilimumab-Tovoralimab	1(4.8%)

### 3.2 Latency and medication

The latency period from ICI initiation to onset of SJS/TEN varied widely, ranging from 1 day to 1 year (median = 28 days). The median number of ICI treatment cycles was four (range, 1–7) (**Table 2**). According to the ALDEN algorithm, five cases scored 4–5 points, and sixteen scored ≥ 6 points. The PD-1 inhibitors used were sintilimab (n = 9), tislelizumab (n = 5), camrelizumab (n = 2), serplulimab (n = 1), and toripalimab (n = 1). Two patients received PD-L1 inhibitors (adebrelimab and atezolizumab), and one received a bispecific PD-1/CTLA-4 antibody (iparomlimab–tuvonrelimab). Additionally, two patients received combination therapy with PD-1 inhibitors (sintilimab or tislelizumab) and the antiangiogenic drug bevacizumab. All patients developed characteristic cutaneous and mucosal manifestations of SJS/TEN, which were confirmed by dermatologists.

**Table 2:** Clinical features of 21 patients with immune checkpoint inhibitor-associated severe cutaneous adverse reactions.

Parameter	Classification	Value
Clinical Classification	Stevens-Johnson Syndrome (SJS)	15 (71.5%)
	SJS/TEN overlap	2 (9.5%)
	Toxic Epidermal Necrolysis (TEN)	4 (19.0%)
Initial Skin Eruption	Erythema (all cases)	21 (100.0%)
	Erythema only	3 (14.3%)
	Mixed morphologies (erythema + papules/scales/blisters)	18 (85.7%)
Progressive Skin Lesions	Erosions	19 (90.5%)
	Epidermal detachment	11 (52.4%)
	Vesicles/Bullae	10 (47.6%)
	Target lesions	6 (28.6%)
	Papules	4 (19.0%)
Mucosal Involvement	Any mucosal involvement	9 (42.9%)
	Oral mucosa (erosions/ulcerations/bleeding/crust)	9/9 (100.0%) of mucosal cases
	Genitourinary mucosa	4 (44.4% of mucosal cases)
	Ocular mucosa	2 (22.2% of mucosal cases)
Systemic Symptoms	Fever	11 (52.4%)
	Median temperature	37.6 °C (range, 37.0–40.0 °C)
Organ Dysfunction	Respiratory Failure	2 (9.5%)
	Acute Kidney Injury	3 (14.3%)
	Hepatic Impairment	4 (19.0%)
Infectious Complications	Skin Infection	4 (19.0%)
	Pulmonary Infection	3 (14.3%)
	Urinary Tract Infection	1 (4.8%)
	Sepsis	1 (4.8%)
Coagulation & Circulatory Abnormalities	Fibrinolytic & Coagulation Dysfunction	5 (23.8%)
	Acute Gastrointestinal Bleeding	1 (4.8%)
Metabolic & Nutritional Disturbances	Hypoalbuminemia	6 (28.6%)
	Electrolyte Imbalance	5 (23.8%)
Hematologic Abnormalities	Bone Marrow Suppression	4 (19.0%)
	Neutropenia	1 (4.8%)
	Anemia	2 (9.5%)
Endocrine Dysfunction	Thyroid Dysfunction	2 (9.5%)
Biopsy Performance	Patients Undergoing Skin Biopsy	10/21 (47.6%)
Epidermal Changes	Epidermal Necrosis/Basal Layer Vacuolar Alteration	6 (60.0%)
	Keratinocytic Necrosis/Apoptosis	5 (50.0%)
	Subepidermal or Intraepidermal Blister Formation	4 (40.0%)
	Epidermal Erosion (variable degree)	4 (40.0%)
Interface/Spongiotic Changes	Interface Dermatitis–like Changes (basal liquefaction ± pigment incontinence)	4 (40.0%)
	Spongiosis	3 (30.0%)
	Acanthosis	3 (30.0%)
Dermal Inflammatory Infiltration	Any Dermal Inflammatory Infiltrate	10 (100.0%)
	Lymphocyte-Predominant Infiltration	10 (100.0%)
	Eosinophils Present	7 (70.0%)
Additional Dermal Findings	Dermal Edema	3 (30.0%)
	Red Blood Cell Extravasation	2 (20.0%)

### 3.3 Clinical manifestations

Among the 21 patients included in this study, the initial skin eruption presented as erythema in all cases (**Table 2**). Of these, 3 patients (14.3%) exhibited only erythema, while the remaining 18 (85.7%) showed mixed morphologies—primarily erythema in combination with papules, scales/desquamation, or blisters. During disease progression, the skin manifestations became more diverse and overlapping in all patients. The most common secondary lesions were erosions (19 cases, 90.5%), followed by epidermal detachment (11 cases, 52.4%) and vesicles/bullae (10 cases, 47.6%). Additionally, target lesions and papules were observed in 6 (28.6%) and 4 (19.0%) patients, respectively. Mucosal involvement was noted in 9 patients (42.9%). The oral mucosa was consistently affected in all 9 cases (100%), manifesting as erosions, ulcerations, bleeding, or crust formation. Genitourinary and ocular mucosal involvement were present in 4 (44.4%) and 2 (22.2%) patients, respectively. Fever occurred in 11 patients (52.4%) with a median temperature of 37.6 °C (range, 37.0–40.0 °C), including two with high fever exceeding 39.0 °C. Based on clinical classification, 15 patients were diagnosed with SJS, 2 with SJS/TEN overlap, and 4 with TEN.

### 3.4 Laboratory findings

Hematologic tests revealed leukocytosis in one patient and leukopenia in three (**Table 2**). Hemoglobin levels were decreased in 15 patients, and thrombocytopenia was noted in two. Inflammatory markers were elevated, with high-sensitivity C-reactive protein increased in 11 and erythrocyte sedimentation rate elevated in 8. Abnormal liver function was observed in several patients: alanine aminotransferase elevation in 5 (including one exceedingly twice the upper limit of normal), aspartate aminotransferase elevation in 4, and gamma-glutamyl transpeptidase elevation in 7. Hypoalbuminemia occurred in 13 patients, suggesting malnutrition or increased protein consumption. Electrolyte imbalances were common, including hyponatremia, hypocalcemia, and hypokalemia. Coagulation tests revealed elevated D-dimer levels in 6 patients and decreased antithrombin activity in 3. Renal function (creatinine, urea nitrogen) remained within normal limits in all cases. Chest CT revealed pneumonia in 3 patients, and one had a positive blood culture.

### 3.5 Acute complications of SJS/TEN

A wide range of acute complications was observed during hospitalization (**Table 2**). Organ dysfunction was common, with respiratory failure occurring in 2 patients, acute kidney injury in 3 patients, and hepatic impairment in 4 patients. Infectious complications were also frequent: skin infections developed in 4 patients, pulmonary infections in 3 patients, and urinary tract infection in 1 patient, while 1 patient progressed to sepsis. Coagulation and circulatory abnormalities included fibrinolytic and coagulation dysfunction in 5 patients and acute gastrointestinal bleeding in 1 patient. Metabolic and nutritional disturbances were notable, with hypoalbuminemia documented in 6 patients and electrolyte imbalance in 5 patients. Hematologic abnormalities included bone marrow suppression in 4 patients, neutropenia in 1 patient, and anemia in 2 patients. In addition, thyroid dysfunction was identified in 2 patients. These findings highlight the substantial systemic involvement and multisystem burden associated with ICI-related SJS/TEN.

### 3.5 Skin biopsy

Skin biopsy was performed in 10 of the 21 patients, and the major histopathological features are summarized in **Table 2**. The most common findings were epidermal necrosis or basal layer vacuolar alteration (6 cases, 60.0%), keratinocytic necrosis or apoptosis (5 cases, 50.0%), and subepidermal or intraepidermal blister formation associated with varying degrees of epidermal erosion (4 cases, 40.0%). Interface dermatitis–like changes, including basal layer liquefaction and pigment incontinence, were observed in 4 cases (40.0%), whereas spongiotic changes and acanthosis were present in 3 cases (30.0%). Inflammatory infiltrates were identified in all biopsies, predominantly lymphocytic infiltration in the superficial or mid-dermis (10 cases, 100%), with eosinophils being the next most frequent inflammatory cell type (7 cases, 70.0%). Focal red blood cell extravasation (2 cases, 20.0%) and dermal edema (3 cases, 30.0%) were also noted. Direct immunofluorescence was performed in 9 patients, all of whom showed negative staining for IgG, IgA, IgM, and C3, except for two specimens demonstrating mild, nonspecific cytoplasmic IgG staining without diagnostic significance.

### 3.7 Treatment and outcomes

All patients discontinued the suspected ICI immediately after diagnosis (**Table 3**). Nineteen patients received systemic corticosteroid therapy at an initial dose of 0.74–1.87 mg/kg/day. The criteria for disease control included darkening of erythema, drying and crusting of blisters, absence of new lesions, appearance of epithelial islands, and a negative Nikolsky sign. If fever or new eruptions persisted after 48–72 hours (excluding infection), corticosteroid doses were increased or combined with intravenous immunoglobulin (IVIG). Eight patients (38.1%) received combination therapy with IVIG. Seven patients received antibiotics for secondary infections involving the respiratory tract or eroded mucocutaneous surfaces. The mean time to cessation of epidermal detachment was 7.8 days (range, 3–15 days), and the mean time to re-epithelialization was 9.7 days (range, 4–18 days).

**Table 3:** Treatment and outcomes of 21 patients with immune checkpoint inhibitor-associated severe cutaneous adverse reactions.

Parameter	Classification	Value
Initial Management	ICI Discontinuation (all)	21 (100.0%)
Systemic Therapy	Systemic Corticosteroids	19 (90.5%)
	Initial Dose (prednisone equivalent)	0.74-1.87 mg/kg/day
	Combination Therapy with IVIG	8 (38.1%)
	Antibiotics for Secondary Infections	7 (33.3%)
Disease Control Metrics	Mean Time to Cessation of Epidermal Detachment	7.8 days (range, 3–15)
	Mean Time to Re-epithelialization	9.7 days (range, 4–18)
Corticosteroid Therapy	Mean Duration	34.2 days (range, 6–150)
Treatment Response	Skin Symptom Improvement	18 (85.7%)
	Median Time to Cutaneous Improvement	26 days (range, 6–150)
	In-hospital Mortality (TEN)	3 (14.3%)
Severity Scoring	SCORTEN = 2	4 (19.0%)
	SCORTEN = 3	12 (57.1%)
	SCORTEN = 4	5 (23.8%)
Hospitalization	Median Length of Stay	15 days (range, 9–42)
Follow-up (Survivors, n=18)	Lost to Follow-up	2 (11.1%)
	Mean Follow-up Duration	6.9 months (range, 1–18)
Oncologic Outcomes	Complete or Partial Remission of Primary Malignancy	0 (0.0%)
	Stable Disease at First Post-discharge Evaluation	10 (62.5% of followed patients*)
	Later Progression After Initial Stability	8 (50.0% of followed patients*)
	Immediate Post-discharge Progression	4 (25.0% of followed patients*)
Post-discharge Mortality	Deaths Due to Tumor Progression/Complications	6 (37.5% of followed patients*)

*Followed patients = 16 (two were lost to follow-up).

The average duration of corticosteroid therapy was 34.2 days (range, 6–150 days). Eighteen patients demonstrated cutaneous improvement, which was defined as cessation of new lesion formation, stabilization of epidermal detachment, and the onset of re-epithelialization over previously denuded areas. Three patients with TEN died from multiple organ failure. The median time to cutaneous improvement was 26 days (range, 6–150 days). SCORTEN scores were 2 in 4 cases, 3 in 12 cases, and 4 in 5 cases. The median length of hospitalization was 15 days (range, 9–42 days). The main cause of death was respiratory failure secondary to pulmonary infection. Among the 18 survivors discharged with improvement, two were lost to follow-up. The remaining 16 patients were followed for a mean of 6.9 months (range, 1–18 months). None achieved complete or partial remission of the primary malignancy. At the first postdischarge evaluation, disease was stable in 10 cases but later progressed in 8 (2 died of terminal malignancy), while 4 exhibited immediate progression (2 died of tumor-related respiratory failure). Another 2 patients died within 1.5 months after discharge due to tumor progression combined with pneumonia and respiratory failure.

## 4 Discussion

In normal immune responses, T-cell activation and inhibition are tightly regulated by multiple immune checkpoints to maintain immune homeostasis and prevent autoimmunity. Tumor cells frequently exploit these inhibitory pathways to escape immune surveillance, among which the cytotoxic T-lymphocyte-associated antigen 4 (CTLA-4) and programmed cell death protein 1 (PD-1) pathways are the most representative. Both CTLA-4 and PD-1 act as negative regulators of T-cell immunity but differ in their sites and timing of action (9, 10). CTLA-4 primarily functions during the early stage of T-cell activation within lymphoid organs by competitively inhibiting CD28-CD80/CD86 interactions, thereby suppressing T-cell proliferation and clonal expansion. In contrast, PD-1 mainly operates during the effector phase in peripheral tissues, where its ligand PD-L1–widely expressed on tumor cells and interferon-γ-stimulated immune cells—transmits inhibitory signals that lead to T-cell exhaustion, reduced cytokine production, and weakened antitumor activity (11–13). Immune checkpoint inhibitors (ICIs) block these inhibitory signaling pathways, effectively releasing the “brakes” on T cells and restoring antitumor immunity. Anti–CTLA-4 antibodies (such as ipilimumab) primarily enhance early T-cell priming, while anti–PD-1/PD-L1 antibodies (such as nivolumab and pembrolizumab) sustain effector T-cell responses within peripheral tissues. These therapies have achieved remarkable and durable efficacy in several malignancies, including melanoma, non–small cell lung cancer, and hepatocellular carcinoma (14, 15).

The precise pathogenesis of ICI-induced SJS/TEN has not been fully elucidated. Goldinger et al. (16) performed immunohistochemical analysis on six patients who developed immune-related cutaneous adverse events (ircAEs) following PD-1 inhibitor therapy. They observed CD8+ T-cell infiltration at the dermo-epidermal junction in all rash tissue samples, along with upregulated expression of PD-1 on lymphocytes and PD-L1 on keratinocytes. The authors proposed that PD-1 inhibitors disrupt the homeostatic PD-1/PD-L1 interaction, thereby inducing severe cutaneous adverse reactions. Research published in Nature further delineates a key pathogenic axis in ICI-induced SJS/TEN, centered on macrophage-derived CXCL10 and TNF signaling. This mechanistic framework also explains why TNF-α inhibitors may demonstrate superior therapeutic effects compared with glucocorticoids–primarily through the disruption of CXCL10-driven CD8+ T-cell activation, thereby preventing blister formation and accelerating epidermal repair (17). Another recent research has established that a shared Th2-dominant immune mechanism underlies the pathogenesis of both lichenoid and eczematous eruptions associated with immune checkpoint inhibitors (18). Ellis et al. (19) further identify that ICI-induced SJS/TEN is characterized by a dominant CD8+ T-cell infiltrate, compensatory PD-L1 upregulation, and a unique cytotoxic/chemokine gene signature, directly linking its pathology to the mechanism of immune checkpoint inhibition.

In this study, all patients with immune checkpoint inhibitor (ICI)–related SJS/TEN discontinued immunotherapy immediately after diagnosis and received comprehensive systemic and multidisciplinary management. The main therapeutic measures included systemic corticosteroids, intravenous immunoglobulin (IVIG), and supportive and symptomatic treatments, such as anti-infective therapy, nutritional support, and maintenance of fluid and electrolyte balance. In addition, some patients were treated with topical corticosteroids or antihistamines to alleviate cutaneous symptoms.

In terms of therapeutic strategies, corticosteroids remain the cornerstone of SJS/TEN management. Although some studies have suggested that high-dose corticosteroid therapy may increase the risk of secondary infections and therefore should be used with caution, an increasing body of evidence supports that early and adequate corticosteroid administration can effectively suppress immune-mediated inflammation, promote epidermal healing, and reduce mortality, without significantly prolonging recovery time (20). Most patients shown marked improvement in cutaneous and mucosal lesions after systemic corticosteroid therapy; for those with insufficient response, adjunctive agents such as cyclosporine, TNF-α inhibitors, or intravenous immunoglobulin (IVIG) can be added to enhance therapeutic outcomes.

Once SJS/TEN is confirmed, immune checkpoint inhibitors should be permanently discontinued, and corticosteroid therapy (prednisone or methylprednisolone, 1–2 mg/kg·d) should be initiated as early as possible. Patients are advised to be managed in an intensive care unit or burn unit under multidisciplinary collaboration involving dermatology, ophthalmology, urology, and otorhinolaryngology. For patients at risk of gastrointestinal bleeding, corticosteroid dosage should be carefully evaluated in close consultation with gastroenterology specialists to balance anti-inflammatory efficacy and hemostatic safety (21, 22).

In addition to corticosteroids, intravenous immunoglobulin (IVIG) represents an important therapeutic option. IVIG can inhibit Fas/FasL-mediated keratinocyte apoptosis, suppress the release of inflammatory cytokines, and exert immunomodulatory effects through toxin neutralization. It is recommended as an adjunctive therapy at a dosage of 0.4 g/kg·d (21, 23, 24). In our cohort, most patients received combined therapy with corticosteroids and IVIG, and several severe cases achieved favorable recovery under multidisciplinary team (MDT) management. Taken together, these observations suggest that early and coordinated intervention with corticosteroids plus IVIG may contribute meaningfully to improved clinical outcomes in patients with ICI-related SJS/TEN (25).

In recent years, targeted therapies aimed at key inflammatory pathways in severe cutaneous adverse reactions have advanced rapidly, providing more controllable immunomodulatory strategies for patients with inadequate responses to systemic corticosteroids or those with underlying malignancies who develop ICI-related SJS/TEN. Cyclosporine, which rapidly suppresses drug-specific T-cell activation, has been shown to shorten re-epithelialization time and reduce mortality, and is now recommended as a steroid-sparing first-line option in several international guidelines (26–29). TNF-**α**inhibitors represent the most well-supported class of biologics; TNF-**α** plays a central role in keratinocyte apoptosis and inflammatory amplification, and multiple studies have demonstrated that etanercept or infliximab combined with systemic corticosteroids significantly shortens re-epithelialization time, reduces gastrointestinal bleeding, and does not increase infectious risk, with similarly favorable outcomes reported in ICI-related SJS/TEN (30–32). Furthermore, JAK inhibitors (e.g., selective JAK1/3 or broader JAK1/2 agents) have shown promising preclinical and early clinical activity in aborting IFN-γ/IL-15–driven cytotoxic programs and accelerating skin recovery in severe cases, although careful monitoring for infectious and thromboembolic risks is required (33–35). Overall, the integration of targeted biologics, JAK inhibitors, and conventional immunosuppressive therapy may allow more precise modulation of pathogenic pathways, accelerated skin repair, and improved outcomes, although their long-term safety and optimal sequencing in the setting of ICI-related SJS/TEN require further validation (36).

Furthermore, special attention should be paid to one patient in our study who developed TEN and ultimately died after receiving the PD-1/CTLA-4 bispecific antibody (Ipilimumab-Tovoralimab, as presented in **Table 1**). This case highlights that simultaneous blockade of the PD-1 and CTLA-4 immune checkpoint pathways may not only potentiate antitumor immunity but also markedly amplify autoreactive immune responses, leading to severe or even fatal toxicities. Previous studies have shown that combination therapy with PD-1/PD-L1 and CTLA-4 inhibitors can induce deeper tumor responses in some patients, but it is also associated with substantially higher rates of severe adverse events, immune-related toxicities, and treatment discontinuation compared with PD-1/PD-L1 monotherapy (37, 38). Extending this therapeutic concept, bispecific antibodies enable dual checkpoint inhibition within a single molecular framework, thereby providing even stronger immune stimulation. However, such intensified immune activation may simultaneously erode self-tolerance, increasing susceptibility to fulminant immune reactions such as SJS/TEN (39).

This fatal case suggests that the toxicity profile of dual immune checkpoint blockade may differ substantially from that of monotherapy, with potential survival benefits accompanied by unprecedented risks. For patients receiving such potent immune combination therapies (including bispecific antibodies or combination regimens), clinicians should perform comprehensive risk assessments before treatment, maintain heightened vigilance throughout therapy, and promptly recognize and manage even mild cutaneous or mucosal symptoms to improve clinical outcomes.

Overall, the clinical outcomes in our cohort–including a 14.3% mortality rate and generally favorable responses to corticosteroids combined with IVIG–align with previous reports of ICI-related SJS/TEN, which similarly describe high SCORTEN scores, substantial morbidity, and rapid disease escalation (40, 41). Our findings further reinforce emerging evidence that dual checkpoint blockade or bispecific antibodies may confer heightened severity, likely due to amplified T-cell activation that lowers immune tolerance and predisposes to fulminant cutaneous injury. Nevertheless, several limitations should be acknowledged. The rarity of ICI-related SJS/TEN resulted in a relatively small sample size, which may restrict generalizability. Skin biopsies were not performed in all patients because of critical illness and concerns regarding infection risk, potentially affecting diagnostic certainty. In addition, the retrospective design and heterogeneity in cancer types, treatment history, and supportive care introduce unavoidable bias and may cloud interpretation of therapeutic efficacy. Despite these constraints, our study provides clinically relevant insights that extend beyond individual patient management. The findings highlight the need to strengthen clinician awareness of severe cutaneous irAEs during ICI therapy and emphasize the importance of robust post-marketing pharmacovigilance systems to promote early recognition, standardized reporting, and timely intervention for rare but catastrophic toxicities. Looking ahead, future research should prioritize the establishment of multicenter prospective registries to improve case capture and refine estimates of incidence, risk factors, and outcomes. Mechanistic studies–such as immunogenomic profiling and microenvironmental characterization of lesional skin–will be critical to elucidate the pathways linking checkpoint inhibition to epidermal necrosis. At the policy level, strategies such as mandatory reporting frameworks, harmonized dermatologic monitoring protocols, and structured guidance for high-risk individuals could further enhance patient safety. Ultimately, a deeper understanding of the clinical trade-off between antitumor efficacy and severe immune-related toxicity is essential. While combination ICIs and bispecific antibodies may yield superior tumor control, they also appear to heighten vulnerability to life-threatening dermatologic reactions. Balancing these competing considerations will require thoughtful patient selection, individualized treatment intensity, and vigilant surveillance–particularly for those receiving intensified checkpoint inhibition.

## 5 Limitations

This study has several limitations. Its single-center, retrospective nature may limit the generalizability of the findings. The small cohort size (n=21) restricts statistical power and precludes meaningful subgroup analyses. In addition, key immunologic biomarkers—including cytokines and cytotoxic mediators implicated in SJS/TEN pathogenesis (42)—were not systematically assessed across all patients. Due to heterogeneous institutional testing practices and the severe condition of many patients at presentation, the available biomarker data were incomplete and insufficient for cohort-level analysis, limiting our ability to investigate mechanistic associations. Furthermore, heterogeneity in treatment regimens and potential biases inherent to retrospective data collection could confound outcomes interpretation. Finally, the relatively short follow-up period may not fully capture long-term prognosis. Future multicenter, prospective studies incorporating standardized biomarker profiling are warranted to validate these findings and improve mechanistic understanding.

## 6 Conclusion

Our findings highlight that early recognition, immediate discontinuation of immunotherapy, and prompt initiation of systemic corticosteroids—often in combination with intravenous immunoglobulin—are critical for improving prognosis. Multidisciplinary management plays a pivotal role, particularly in severe cases, to minimize complications and mortality. Given the increasing use of immune checkpoint inhibitors, clinicians should maintain high vigilance for severe cutaneous adverse reactions and differentiate them from other immune-related events at an early stage. Future studies are warranted to elucidate the immunopathogenic mechanisms of ICI-induced SJS/TEN and to identify predictive biomarkers that can guide personalized prevention and management strategies.

## Data Availability

The original contributions presented in the study are included in the article/supplementary material. Further inquiries can be directed to the corresponding author/s.
