# Correction: Immune checkpoint inhibitor-related Stevens-Johnson syndrome and toxic epidermal necrolysis: a retrospective analysis of 21 cases

**DOI:** 10.3389/fimmu.2026.1804871

**Published:** 2026-02-16

**Authors:** Tianshu Pu, Yaru Teng, Yuezhu Zhang, Meihong Da, Fei Wang

**Affiliations:** 1 Medical College, Southeast University, Nanjing, China; 2 Department of Dermatology, ZhongDa Hospital Southeast University, Nanjing, China

**Keywords:** cutaneous adverse reactions, dermatology, drug eruptions, ICIS, immune checkpoint inhibitors, immune-related adverse events, severe drug eruptions, Stevens-Johnson Syndrome

“Tianshu Pu^1†^, Yaru Teng^1,2†^, Yuezhu Zhang^1,2^, Meihong Da^2^, Fei Wang^1,2*^

1.Medical College, Southeast University, Nanjing, China

2.Department of Dermatology, Zhongda Hospital Southeast University, Nanjing, China.”

Author “Tianshu Pu.” was erroneously assigned to affiliation “Department of Dermatology, ZhongDa Hospital Southeast University, Nanjing, China”. This affiliation has now been removed for author “Tianshu Pu”.

In the published article, there was a mistake in the **Funding** statement. The **Funding** statement was displayed as “The author(s) declared that financial support was not received for this work and/or its publication.”. The correct statement is “Zhongda Hospital Southeast University, Jiangsu Province HighLevel Hospital Construction Funds, 2024GSPKY18; Basic Research Program-Natural Science Foundation-Youth Fund Project, Project Number: BK20230838”.

The original version of this article has been updated.

